# Relationship between autism and brain cortex surface area: genetic correlation and a two-sample Mendelian randomization study

**DOI:** 10.1186/s12888-024-05514-8

**Published:** 2024-01-23

**Authors:** Xianjing Li, Miaomiao Jiang, Liyang Zhao, Kang Yang, Tianlan Lu, Dai Zhang, Jun Li, Lifang Wang

**Affiliations:** 1 NHC Key Laboratory of Mental Health (Peking University), National Clinical Research Center for Mental Disorders (Peking University Sixth Hospital, Peking University Sixth Hospital, Peking University Institute of Mental Health, Beijing, China; 2 Guangdong Key Laboratory of Mental Health and Cognitive Science, Institute for Brain Research and Rehabilitation (IBRR), South China Normal University, Guangzhou, China

**Keywords:** Autism spectrum disorder, Cortical surface area, Mendelian randomization analysis, Genetic correlation

## Abstract

**Background:**

Alterations in surface area (SA) in specific regions of the cortex have been reported in many individuals with autism spectrum disorder (ASD), however, the genetic background between ASD and SA is still unclear. This study estimated the genetic correlation and causal effect of ASD and cortical SA.

**Methods:**

Summarized data of genome-wide association studies (GWAS) were separately downloaded from the Psychiatric Genomics Consortium (18,381 cases of ASD, and 27,969 controls) and the Enhancing Neuroimaging Genetics through Meta-Analysis Consortium (33,992 participants of Europeans). We used Linkage disequilibrium score regression (LDSC) and Heritability Estimation from Summary Statistics (HESS) to calculate the heritability of each trait. As for the genetic correlation between ASD and SA, LDSC was used for global correlation and HESS was used to examine the local genetic covariance further. We used three Mendelian randomization (MR) methods, Inverse-variance weighted, MR-Egger, and weighted median to estimate the causal relationship.

**Results:**

LDSC observed a nominal significant genetic correlation (rg = 0.1229, *P*-value = 0.0346) between ASD and SA of the rostral anterior cingulate gyrus whereas analysis through HESS did not reveal any significant loci having genetic covariance. Based on MR results, statistically meaningful estimations were found in the following areas, postcentral cortex (β (SE) = 21.82 (7.84) mm, 95% CI: 6.46 to 37.19 mm, P_IVW_ = 5.38 × 10^− 3^, P_FDR_ = 3.09 × 10^− 2^), posterior cingulate gyrus (β (SE) = 6.23 (2.69) mm, 95% CI: 0.96 to 11.49 mm, P_IVW_ = 2.05 × 10^− 2^, P_FDR_ = 4.26 × 10^− 2^), supramarginal gyrus (β (SE) = 19.25 (8.43) mm, 95% CI: 29.29 to 35.77 mm, P_IVW_ = 2.24 × 10^− 2^, P_FDR_ = 4.31 × 10^− 2^).

**Conclusion:**

Our results provided genetic evidence to support the opinion that individuals with ASD tend to develop differences in cortical SA of special areas. The findings contributed to understanding the genetic relationship between ASD and cortical SA.

**Supplementary Information:**

The online version contains supplementary material available at 10.1186/s12888-024-05514-8.

## Background

Autism spectrum disorder (ASD) is a group of neurodevelopmental disorders. Considered a chronic disease, ASD is childhood-onset and will last a lifetime. The core symptoms of ASD include social communication disorders, interaction disorders, and stereotyped behaviors [1]. For people diagnosed with ASD, males have a higher prevalence than females [2]. With more than 1% prevalence in the world [3], ASD greatly increases the burden of mental disorders [4].

Most previous anatomical studies of the brain in ASD have analyzed cortex volume [5]. Surface area (SA) is one of the important factors affecting volume and can provide important information for neurobiological studies of ASD. A meta-analysis of twin studies found that SA was highly heritable [6]. By measuring SA, different aspects of the underlying neural structure can be reflected. For example, according to the Radial Unit Hypothesis, the cerebral cortex develops during embryogenesis as an array of interacting cortical columns, or ‘radial units’, each of which originates from a transient stem cell layer called the ventricular zone [7]. The hypothesis suggests that the number of cortical columns determines the size of the cortical SA while the number of columns depends on the rate of cell proliferation and programmed cell death in the ventricular zone.

Therefore, it is necessary to explore the changes in SA of the brain cortex to better understand the neurological mechanism related to ASD brain abnormalities for future cause studies. Even though there has been much research on the specific differences in the SA area of individuals with ASD, the results of the existing research reports are inconsistent. Some studies found no significant alternation of cortical SA between groups with ASD and control groups after statistic correction. For example, research from Daniel et al. discovered several markers at cortical thickness, volume, and gyrification, but not at SA, based on Magnetic Resonance Imaging (MRI) results composed of participants of 60 individuals with ASD and 41 matched typically developing subjects [8]. Also, in a study targeting brain overgrowth in ASD, no significant SA difference was found by MRI data from 64 ASD individuals and 64 control subjects [9]. Other studies gave different opinions on SA changes in the ASD group. For instance, in a study composed of MRI scan data composed of 121 participants(60 ASD cases and 61 controls), decreased SA was observed in the fusiform gyrus and the middle temporal gyrus [10]. As for research from Patriquin et al. with 115 participants (55 ASD cases and 60 controls), SA alternation was found in the insula and fusiform of the brain cortex [11].

Although much observation research has discussed the correlation between ASD and SA of the brain cortex, there are many inconsistencies in their results and limitations in the research process. The reason for limitations might be caused by fewer people incorporated in research, which could lead to insufficient statistical robustness, and there could be bias caused by different population characteristics. In addition, according to the opinion of George et al., as for various mixed factors or reverse factors, the correlation between risk factors and results could not be reasonably interpreted [12]. Therefore, other methods are needed to study the relationship between ASD and SA of the brain.

Linkage disequilibrium score (LDSC) regression and Mendelian randomization (MR) analyses are two methods that can reveal the associations between ASD and SA. LDSC can assess SNV-based phenotypic heritability and calculate the genetic correlation between two traits [13]. The method proposed for estimating genetic correlation from summary statistics relies on the fact that the genome-wide association studies (GWAS) effect size estimate for a given SNP includes the effects of all SNPs that are in linkage disequilibrium with that SNP. Similar to LDSC, Heritability Estimation from Summary Statistics (HESS) is also a statistical method used to estimate the genetic correlation between two traits. HESS can be used to further examine local genetic correlation [14, 15].

MR uses genetic variation specifically associated with an estimated exposure as an instrumental variable to make inferences about the causal effect of the exposure on the outcome [16, 17]. Estimates from MR are less influenced by environmental confounders because the distribution of genetic variation associated with a particular exposure is largely independent of factors that confound the exposure-outcome association in traditional observational analyses. Furthermore, since an individual’s genotype is determined at the moment of fertilization and remains unchanged by subsequent disease outcomes, the direction of causality is always from genetic variation to the desired trait, thus excluding the possibility of reverse causation.

In this study, we used summary-level data of GWAS from quite large samples for ASD and SA to estimate their genetic background links between the traits. Then we used a Two-sample MR analysis to investigate the causal effect of exposure on the outcome.

## Methods

**Fig. 1:**
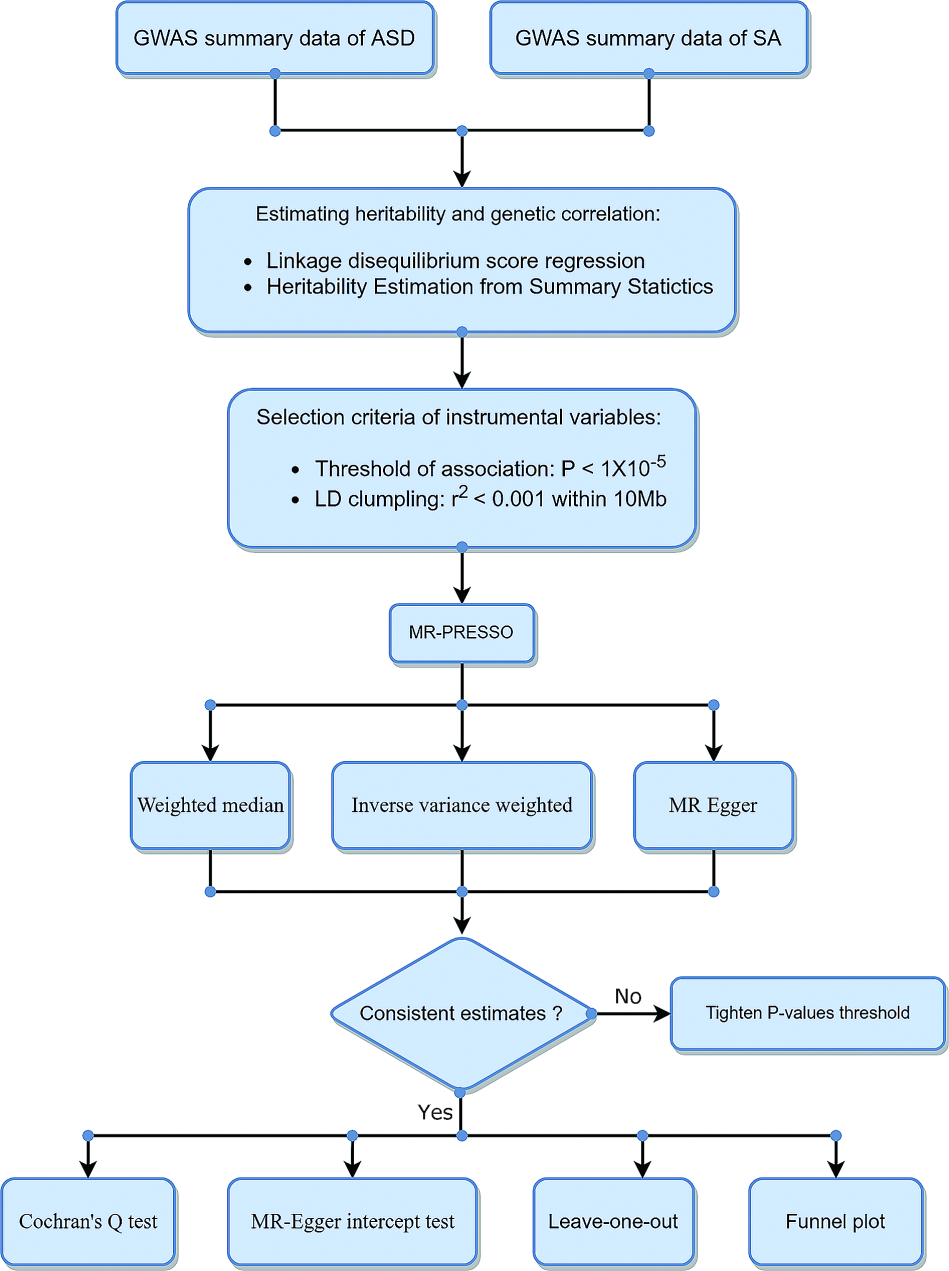
Flowchart of our genetic correlation and Mendelian randomization analysis ASD, autism spectrum disorder; SA, surface area

We estimated the heritability and genetic correlation by approaches of LDSC and HESS and then conducted a two-sample MR to investigate the causal associations of these selected brain regions with ASD. The study flowchart is presented in Fig. 1.

### Genetic data sources for ASD

Genetic summarized data on ASD were downloaded from the Psychiatric Genomics Consortium (PGC) (https://pgc.unc.edu/for-researchers/download-results/), which contained GWAS data on kinds of mental illness. There were 18,381 cases of ASD, and 27,969 controls [18].

### Data sources for brain cortical phenotype

We searched articles related to autism and brain surface area in Pudmed. Brain regions that had been reported more than once to be associated with ASD were included in the study (see brain regions included in the study and their corresponding literature in Supplementary Table S1).

To identify genetic loci associated with human cortical variation, Katrina et al. performed a genome-wide association meta-analysis of cortical SA measurements in 51,665 individuals, predominantly (approximately 94%) of European ancestry, from 60 cohorts around the world. Cortical SA measurements were derived from processing software in vivo whole-brain T1-weighted MRI scans using FreeSurfer MRI-processing software [19, 20]. These cohorts included different gender and age groups. The largest data sources in the meta-analysis included the ENIGMA consortium and the UK Biobank. Our study used meta-results that included only participants of European ancestry, and the sex and age composition of the included cohorts were presented in Supplementary Table S2. The brain cortical GWAS summarized-level data from this meta-analysis were open-accessed from the Enhancing Neuro Imaging Genetics consortium Through Meta-Analysis (ENIGMA) (https://enigma.ini.usc.edu/). The cerebral cortex was divided into 34 regions according to the Desikan-Killiany atlas [21]. In our study, we only paid attention to the selected cortical regions. Because we analyzed different area data separately, the set not weighted by the whole brain was chosen as outcome data.

### Genetic correlation of LDSC and HESS

The level of LD score can measure the degree of genetic variation. With the method of LDSC, influences by SNPs can be estimated [22, 23]. Here, we used LDSC to estimate heritability for ASD, each phenotype of brain cortex SA, and their genetic correlations. Since the summary-level GWAS data including ASD and SA of the brain cortex were from Europe, we used European LD Scores estimations calculated from 1000 Genomes data as the panel to conduct LDSC.

Like LDSC, HESS is also a software package for estimating genetic covariance (correlation) from GWAS summary association data. We used HESS (0.5.4-beta) as a supplement method for further analysis to calculate the local genetic covariance. According to the recommendation of the tutorial on the official website(https://huwenboshi.github.io/hess/), we used the approximately LD-independent loci of European provided by Berisa et al., in which genome was divided into 1703 independent regions. This regional division method was proposed by Tomaz Beris for choosing segment boundaries [24]. The rationale for dividing the genome into these regions was to identify regions of the genome that were associated with complex traits. Using LD-aware breakpoints could avoid stretches of SNPs in LD, which would result in the double-counting of an association signal. The 1000 Genomes reference panel for Europeans was used as a Reference panel.

### Selecting genetic instruments for MR

We used uniform criteria to achieve genetic instruments from ASD GWAS for screening genetic instruments. All picked SNPs were satisfied *P* ≤ 1 × 10^− 5^. A looser threshold is acceptable while only a few genome-wide associated SNPs satisfy this threshold *P* < 5 × 10^− 8^ [25, 26]. R package “ieugwasr” was used for local clumping with a linkage disequilibrium [LD] (r^2^ < 0.001 within 10 Mb). A reference panel of Europeans can be achieved from MRC IEU software (http://fileserve.mrcieu.ac.uk/ld/1kg.v3.tgz) [27]. SNPs with a threshold of *P* ≤ 1 × 10^− 5^, were considered associated with outcome data and then removed based on the exclusionary assumption of Mendelian randomization. F statistic > 10 is considered sufficient to provide sufficient information for MR analysis [28, 29].

### MR analysis

Before each MR analysis, MR Pleiotropy RESidual Sum and Outlier (MR-PRESSO) was used to eliminate the outlier instrumental variables. We used three methods: Inverse variance weighted (IVW), Weighted median, and MR Egger, to estimate the relationship between ASD and SA of the brain cortex. These three MR methods, IVW, Weighted median, and MR Egger can be conducted by R package “TwoSampleMR”. Among them, IVW results are the main reference results. If the direction of β values of these three analyses is inconsistent, a tightened *p*-value is needed for another MR analysis. Cochron’s Q TEST was used to estimate heterogeneity while funnel plot was used to assess the probable directional pleiotropy. If the *p*-value of Cochran’s Q test is less than 0.05, the results are considered to be heterogeneous, which indicates that the causal effect of the exposure on the outcome is not consistent across different subgroups. This could be due to differences in genetic background, environmental factors, or other factors that influence the relationship between the exposure and the outcome [30]. MR-Egger intercept test and leave-one-out analysis were used to detect horizontal pleiotropy and directional pleiotropy. For all IVW results of MR analysis, we calculate a false discovery rate (FDR) correction for multiple comparisons [13, 31], which could be achieved by the R package “fdrtool”. The statistical method FDR, also called the BH rule, was brought by Benjamini and Hochberg, to control false positive rates [32].

## Results

### Genetic correlation between ASD and SA

**Fig. 2:**
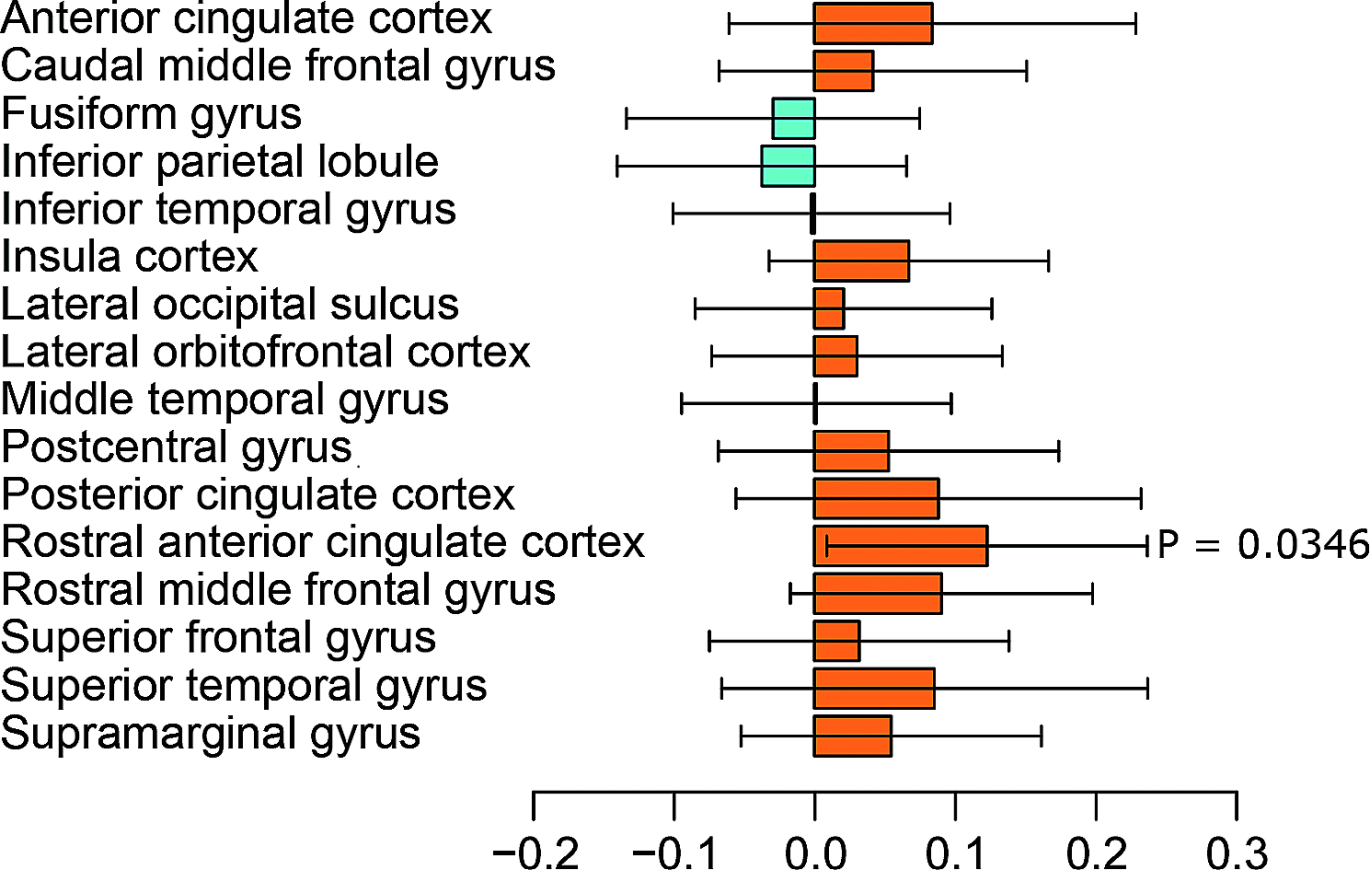
Genetic correlation estimates of Linkage disequilibrium score regression

According to results from LDSC, heritability for ASD was 0.194. Results for bivariate LDSC identified a nominal significant genetic correlation between ASD and rostral anterior cingulate gyrus (rg = 0.1229, *P*-value = 0.0346), which failed the multiple corrections of FDR. The genetic correlation between ASD and SA was shown in Fig. 2.

Results of heritability for each region were shown in Supplementary Table S3, in which SNP heritability h² for postcentral, posterior cingulate, and supramarginal were 25.0% (standard error (SE) = 2.1%), 20.1% (SE = 2.2%) and 21.9% (SE = 2.1%) when estimated using LDSC respectively. As for the results of HESS, heritability for ASD was 0.159, slightly different from the result of LDSC. SNP heritability h² for postcentral, posterior cingulate, and supramarginal were 8.2% (SE = 3.2%), 6.8% (SE = 3.2%), and 8.2% (SE = 3.2%) when estimated using HESS respectively (Table S4). We also performed estimations of local genetic covariance by HESS and identified weak genetic covariances between ASD and cortex SA (Table S4). We visualized local SNP heritability estimates and local genetic covariance estimates via Manhattan plots, in which colored bars represented loci that had significant local SNP heritability or that had significant local genetic covariance. In the Manhattan-style plots of genetic covariance analysis between ASD and cortex SA, all bars were gray indicating that no loci exhibited significant local genetic covariance (Fig. S1).

### Causal estimates of ASD on SA of brain cortex area

In total, 45 instrumental variables of SNPs were picked out for ASD. F statistical values of these instrumental variables varied between 19.5 and 35.7, indicating sufficient information for MR analysis (see more information on index SNPs in Supplementary Table S5). For MR-presso estimates, the *p* values of the global test were all > 0.05, indicating there were no outliers among the instrumental variables (results of MR-presso were shown in Supplementary Table S6). The estimates of these three MR approaches were inconsistent in the analysis of fusiform, inferior parietal gyrus, inferior temporal gurus, and lateral occipital gyrus. Therefore, a tightened *p*-value threshold was needed for reanalyzing. After using a tightened *p*-value threshold, the *P*-values of IVW were greater than 0.05, indicating that there was no potential causal relationship between ASD and these cortical SA (Supplementary Table S7).

**Fig. 3:**
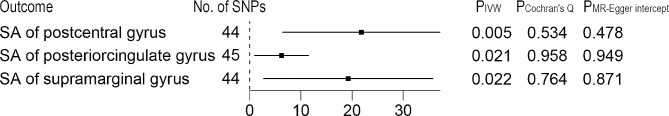
The causal effect of genetically predicted ASD on cortical SA ASD, autism spectrum disorder; SA, surface area; SNP, single-nucleotide polymorphism; IVW, inverse-variance weighted

**Fig. 4:**
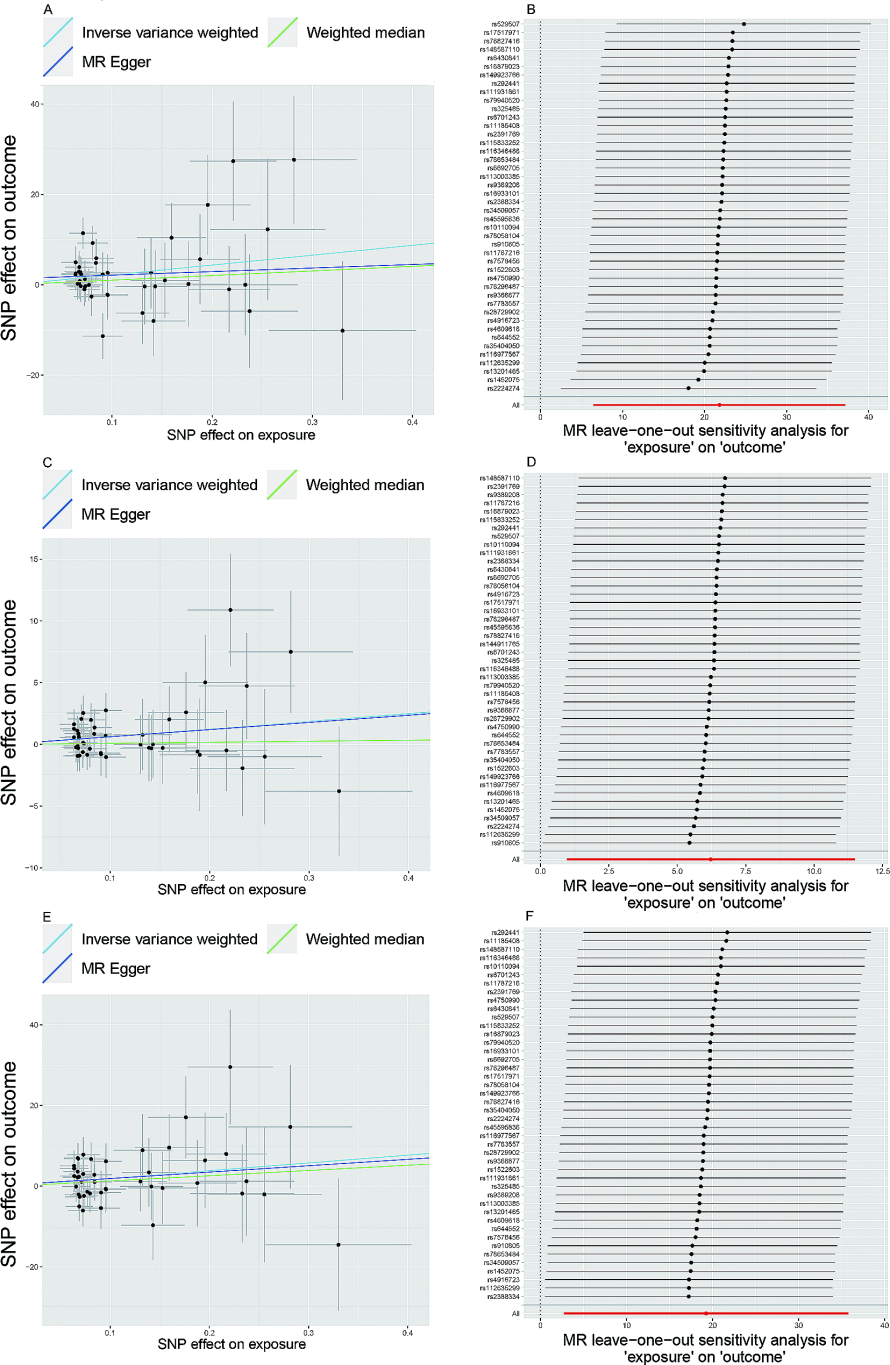
Scatterplot of single-nucleotide polymorphism (SNP) associated with ASD and cortical surface area. **A**: Scatterplot of SNP associated with ASD versus the cortical SA of postcentral. **B**: Leave one out regression analysis of ASD versus the cortical SA of postcentral. **C**: Scatterplot of SNP associated with ASD versus cortical SA of posterior cingulate. **D**: Leave one out regression analysis of ASD versus the cortical SA of the posterior cingulate. **E**: Scatterplot of SNP associated with ASD versus cortical SA of supramarginal. **F**: Leave one out regression analysis of ASD versus the cortical SA of supramarginal ASD, autism spectrum disorder; SA, surface area. Vertical and horizontal lines around each SNP show a 95% confidence interval for the scatterplot

The results surviving from multiple adjustments of FDR (Supplementary Table S8), revealed that ASD was associated with the increased SA of the postcentral cortex (β (SE) = 21.82 (7.84) mm, 95% CI: 6.46 to 37.19 mm, P_IVW_ = 5.38 × 10^− 3^, P_FDR_ = 3.09 × 10^− 2^), posterior cingulate gyrus (β (SE) = 6.23 (2.69) mm, 95% CI: 0.96 to 11.49 mm, P_IVW_ = 2.05 × 10^− 2^, P_FDR_ = 4.26 × 10^− 2^), supramarginal gyrus (β (SE) = 19.25 (8.43) mm, 95% CI: 29.29 to 35.77 mm, P_IVW_ = 2.24 × 10^− 2^, P_FDR_ = 4.31 × 10^− 2^ (see Fig. 3). Scatter plots showed the MR effect of each exposure on ASD. The slope value represented the causal effect. The three MR analysis estimates shown in the figure were consistent in direction (see Fig. 4A, C, and E).

Results of MR Egger and weighted median were presented in Supplementary Fig. S2. Among the three analytical methods employed, only the IVW yielded a *P*-value less than 0.05, indicating statistical significance. When selecting analysis methods for MR, three principles should be considered [33–35]. In the absence of heterogeneity and pleiotropy, IVW estimation results are preferred. When there is heterogeneity but no pleiotropy, the results of the Weighted Median method are preferred (random-effects models of IVW can also be used). When there is pleiotropy, the results calculated by the MR-Egger method are preferred. In our study, we first used the MR-PRESSO package to detect the presence of outliers in the data. If outliers were detected, we removed them and proceeded with the analysis. We also conducted the leave-one-out sensitivity analysis method to test for outliers. To ensure the reliability of the final analysis results, the direction of the analysis results (β value) was consistent among the three main methods. Consequently, we used IVW as the primary outcome.

For these meaningful estimates of IVW, there was no significant result (*p*-value > 0.05) in the MR-Egger intercept test, indicating there was no horizontal pleiotropy (see Supplementary Table S9). As for Cochran’s Q test, there was no heterogeneity between these instrumental variables with a *p*-value > 0.05 (see Supplementary Table S9). Results of the leave-one-out analysis showed that deleting specific SNPs did not cause a significant change, which indicated these SNPs did not have a significant impact on the outcome variable (see Fig. 4B, D, and F). The funnel plots were symmetric as a whole, and there was no obvious deviation point (see Supplementary Fig. S3). Also, we performed the Mendelian randomization analysis using cortical SA as exposure. The analysis results were showed in the supplementary table S10. Among them, the *P* values of IVW were all greater than 0.05, indicating that there was no reverse causality.

## Discussion

In this study, we estimated the genetic correlation between SA and ASD, while LDSC showed nominal significant results in the rostral anterior cingulate gyrus (rg = 0.1229, *P*-value = 0.0346). Through MR analysis, several brain cortex SA was found to tend to increase with *p* values < 0.05 after FDR correction, including the postcentral gyrus, posterior cingulate gyrus, and supramarginal gyrus. Our results provided genetic evidence to support the opinion that individuals with ASD tend to develop differences in cortical SA of special areas.

Dysfunction of the rostral anterior cingulate gyrus was found associated with Social Disability [36]. One study by Keith M. Shafritz et al. linked this region to repetitive behaviors in ASD [37]. In this study, repetitive behavior was inversely associated with fMRI activation in the rostral anterior cingulate gyrus during the experiment. Similarly, research from Katharine N. Thakkar et al. found that impairment of structure and function would lead to repetitive and stereotyped behaviors in individuals with ASD [38]. The postcentral gyrus, where the somatosensory cortex is located, not only plays a crucial role in processing sensory information from other parts of the body, but also has a significant influence on emotional processing, including identification of emotional significance in a stimulus, generation of emotional states, and regulation of emotion [39]. It was reported that individuals with autism had emotional defects [40], which would lead to challenges encountered in social interaction in social interaction. Many mental diseases associated with these regions have been reported, such as major depression and bipolar disorder [39]. The posterior cingulate cortex has an important role in cognition, while cognitive developmental impairment is one of the major autistic symptoms [41]. The region also shows abnormalities in other neurological and psychiatric disorders including Alzheimer’s disease, schizophrenia, autism, depression, attention deficit hyperactivity disorder, and aging [42]. The supramarginal gyrus was studied and associated with phonological processing and verbal working memory [43], also its activation was shown to relate to higher expressive language [44]. The defect of voice memory is a characteristic of people with ASD and might affect their language acquisition [45]. The radial unit hypothesis proposes that the expansion of cortical surface area is driven by the proliferation of neural progenitor cells [46]. Whether the altered surface area of the autism cortex is related to the abnormal proliferation of neural progenitor cells needs further study.

Summarized GWAS data used in this study were from considerably large samples, which usually led to higher levels of statistical effects. This research used two analysis methods, LDSC and HESS, to estimate the genetic relationship based on summarized GWAS data. In general, their estimates should be similar. The differences between estimates might be caused by the following reasons. HESS and LDSC have different definitions of genetic covariance. LDSC uses full genome LD scores, whereas HESS uses local LD information [15]. LDSC assumes that the contribution of each SNP to the phenotype was independent, and HESS allowed interaction between SNP [47]. We used MR to explore whether there was a causal relationship between ASD and the change of surface area in the brain cortex. MR is one of the most powerful genetic epidemiological methods. The instrumental variables we chose were closely related to ASD and are independent of exposure, also F statistics were all larger than 10. To keep the consistency of the direction for MR results, we used three methods including IVW, MR-Egger, and Weighted median. To ensure the reliability of the results, we also performed heterogeneous and sensitive analyses for each result.

Although this study corrected the results of IVW multiple times, it still had limitations. In our study, the IVW method was the only one to yield a *P*-value less than 0.05, indicating a robust and reliable association between ASD and cortex SA in the absence of heterogeneity and pleiotropy. However, the non-significant results from the MR Egger and Weighted Median methods suggested that these methods may not have enough power to detect an association in this particular cortex. These results indicated the complexity of the relationship between ASD and SA and highlighted the need for larger datasets in future research.

Our study had some additional limitations [35, 48]. Firstly, all enrolled participants were European, therefore, a causal relationship between ASD and SA in other populations remains unknown. Secondly, the genetic instruments associated with ASD were from GWAS in which ASD was considered a binary trait, representing the average causal effect in MR analysis [49]. In addition, our outcome data were derived from MRI results belonging to different study cohorts, which were also influenced by several methodological factors related to differences in anatomical sex, intelligence quotient, and age-dependence of neurodevelopment. Finally, more large-scale GWAS data are needed and the underlying mechanism of the altered cortical structure in ASD deserves further study, especially some periods of particular significance, such as infancy within the first 2 years and the transition from childhood to adolescence [50, 51].

While efforts to identify consistent differences in the brains of individuals with ASD remain inconclusive based on previous studies, there is a growing need for brain-based predictive markers [52]. Our research used large summarized GWAS data across LDSC, HESS, and MR methods to estimate genetic associations and casual relationship between ASD and SA of the brain cortex, and reported that individuals with ASD tend to have larger SA in several regions, which could provide candidate biomarkers for ASD diagnosis and new insights of explanations for its related symptoms.

## Conclusions

This study used summarized data of GWAS to conduct genetic correlation estimation and MR analysis to reveal the genetic background between ASD and cortical SA. Our estimates suggested that ASD causally increases the cortical SA of postcentral, posterior cingulate, and supramarginal. This study helped explore and discover the genetic relationship between ASD and cortical SA. The mechanisms underlying the link between ASD and changes in cerebral cortex structure and function deserve further investigation.

## Electronic supplementary material

Below is the link to the electronic supplementary material.

**Supplementary Material 1: Supplementary Figure 1.** Local genetic covariance estimates of Heritability Estimation from Summary Statistics. **Supplementary Figure 2.** Forest plot of significant estimates identified with IVW. **Supplementary Figure 3.** Funnel plot from genetically predicted ASD on SA

**Supplementary Material 2: Table S1.** Information of studies on SA alternation between ASD and controls. **Table S2.** Descriptions of study cohorts from ENIGMA3 Cortical GWAS Data. **Table S3.** The results of LDSC for each cortical SA. **Table S4.** The results of HESS for each cortical SA. **Table S5.** 45 index SNPs represented genetically predicted ASD. **Table S6.** Results of MR-presso. **Table S7.** Details for Mendelian randomization studies with tightened instrument variables selection. **Table S8.** Results of IVW through FDR correction. **Table S9.** Results of MR-Egger intercept test and Cochran’s Q test. **Table S10.** Results of Mendelian randomization with the cortical surface area as the exposure

## Data Availability

All of the summary-level GWAS data used in this study are open-accessed. Data on ASD can be downloaded from the Psychiatric Genomic Consortium (PGC: https://pgc.unc.edu/for-researchers/download-results/) and data on the brain can be downloaded from Enhancing Neuro Imaging Genetics Through Meta-Analysis (ENIGMA: https://enigma.ini.usc.edu/). Computing code can be available through corresponding authors.
