# Supplementary material for: Relationship between autism and brain cortex surface area: genetic correlation and a two-sample Mendelian randomization study
Source: BMC Psychiatry. 2024 Jan 23;24:69. doi: 10.1186/s12888-024-05514-8 (PMC10807092; doi:10.1186/s12888-024-05514-8)

**Supplementary Figure 1** Local genetic covariance estimates of Heritability Estimation from Summary Statistics

ASD, autism spectrum disorder; SNP, single-nucleotide polymorphism.


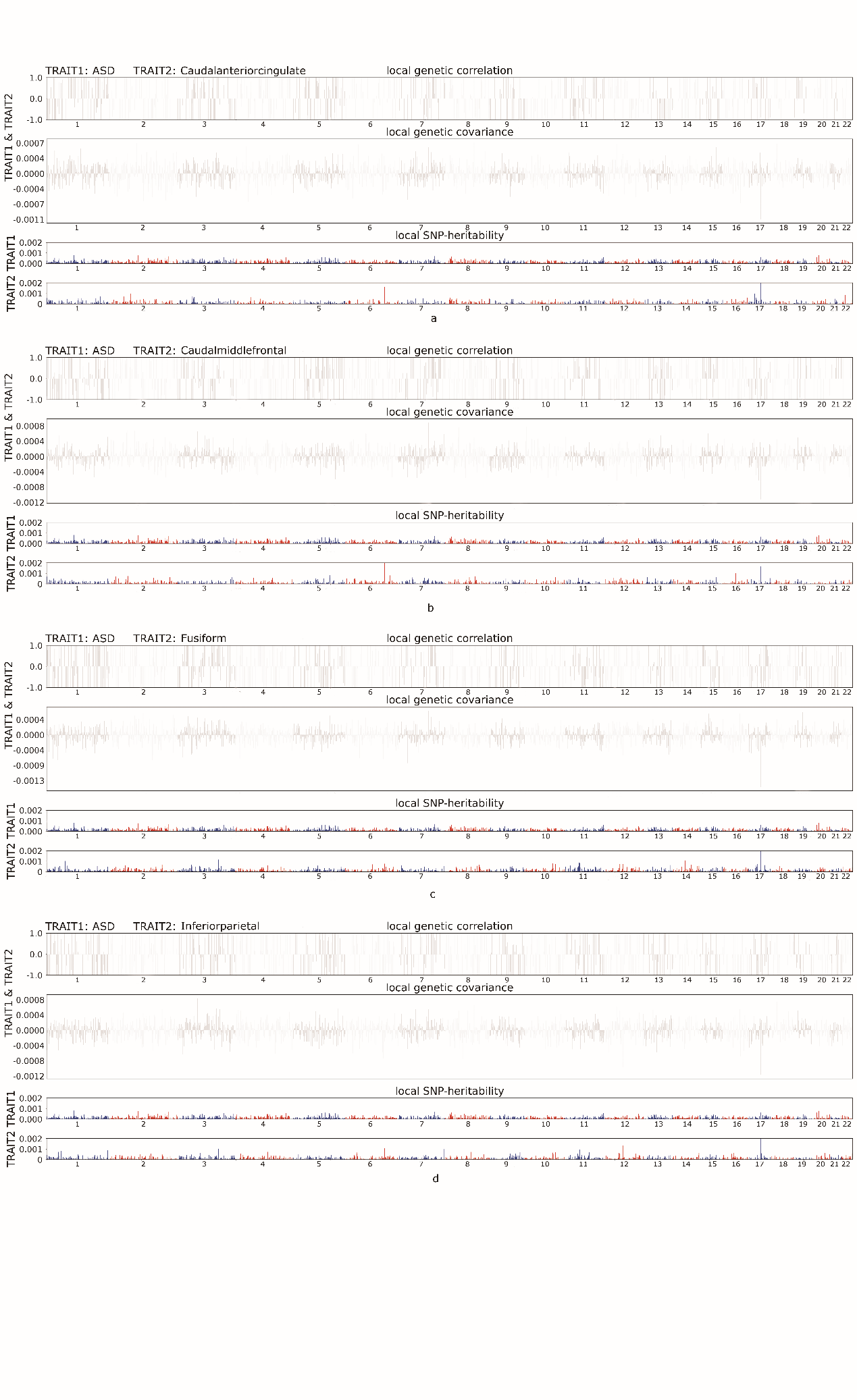


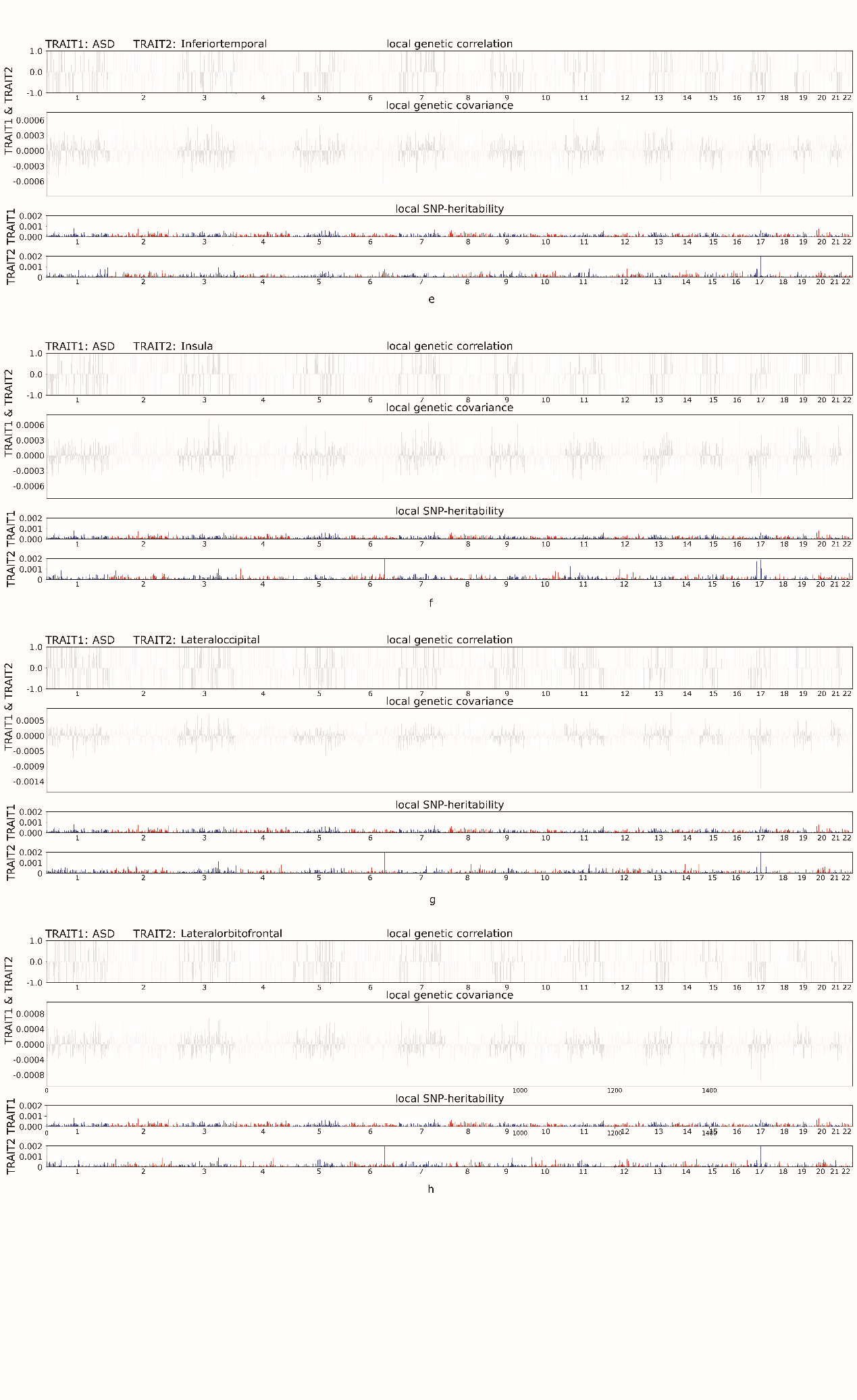


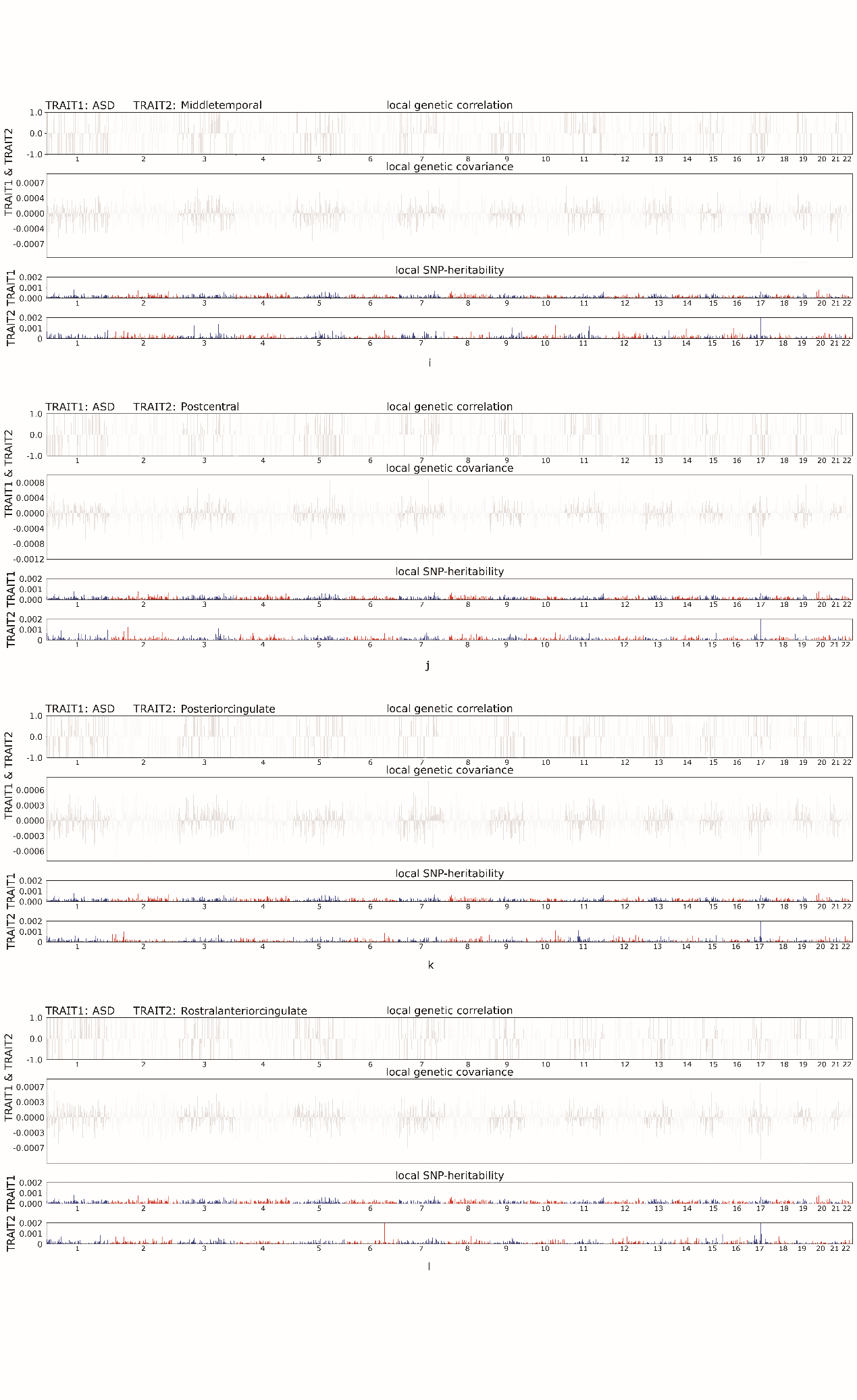


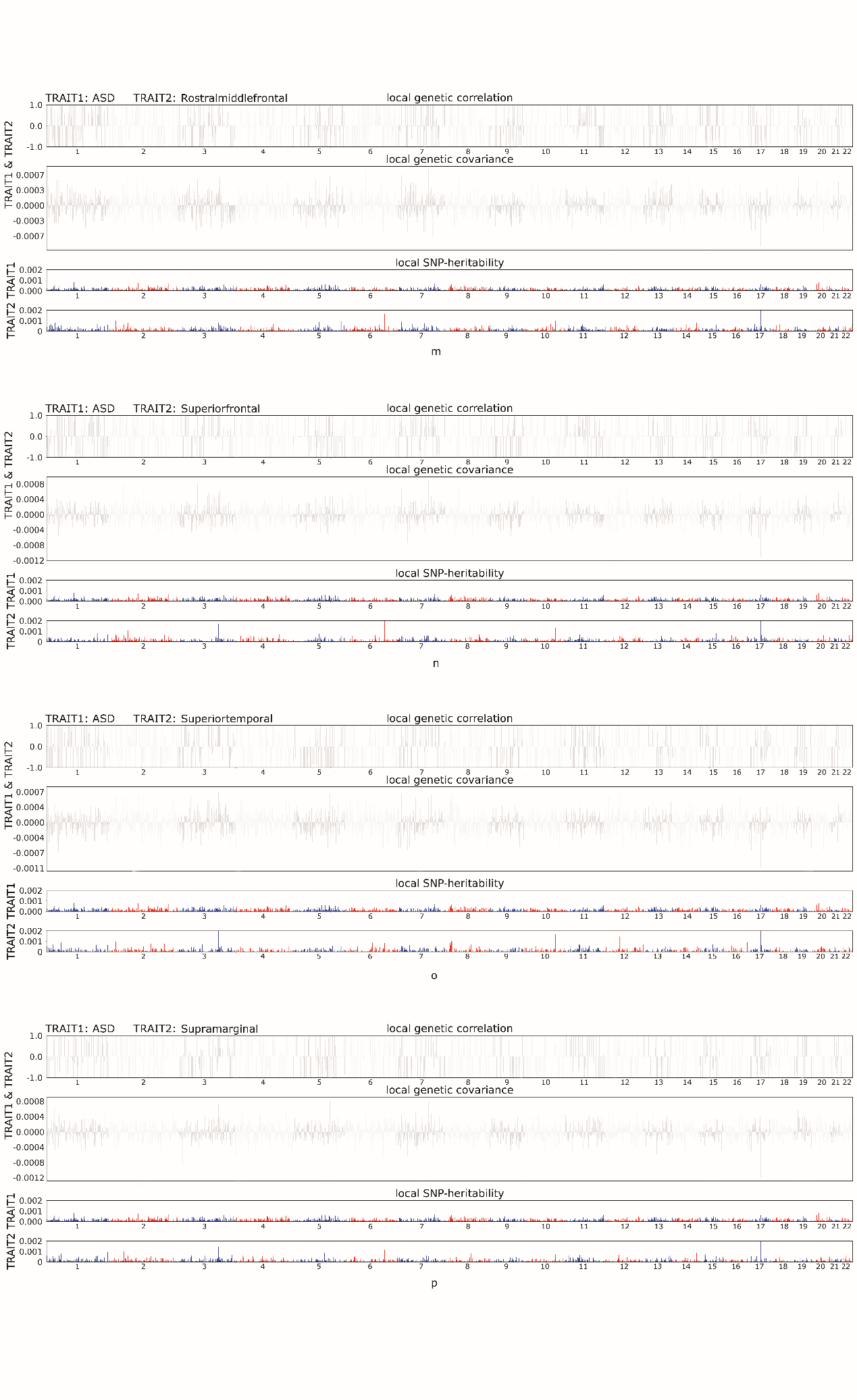


**Supplementary Figure 2** Forest plot of significant estimates identified with IVW

IVW, inverse-variance weighted.


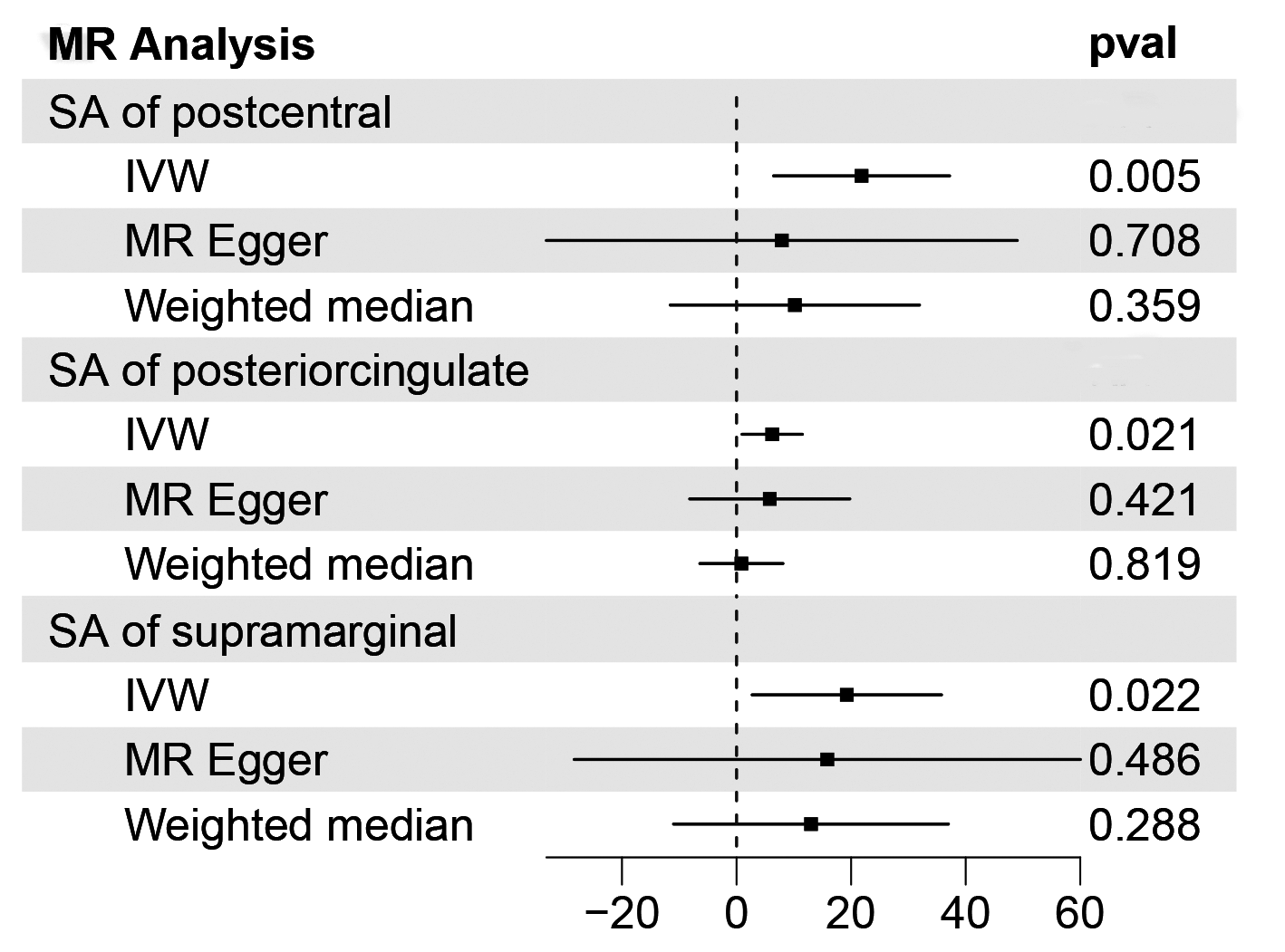


**Supplementary Figure 3** Funnel plot from genetically predicted ASD on SA

a surface area of the postcentral. b surface area of the posterior cingulate. c the surface area of the supramarginal.

ASD, autism spectrum disorder; SA, surface area.


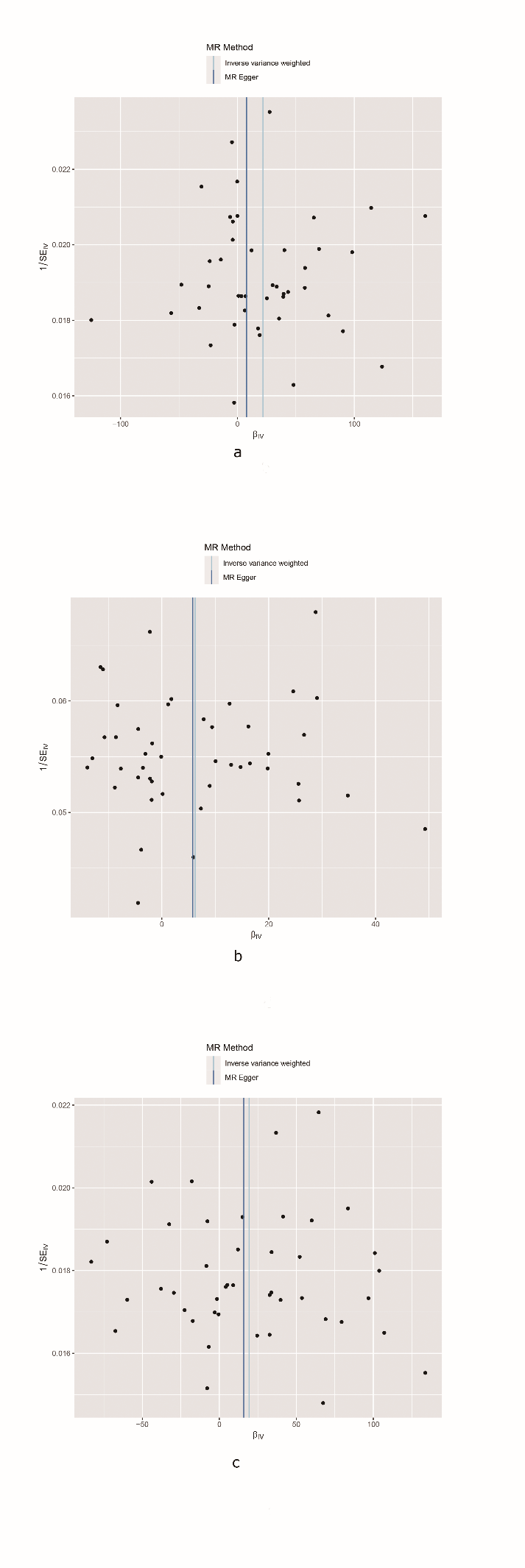

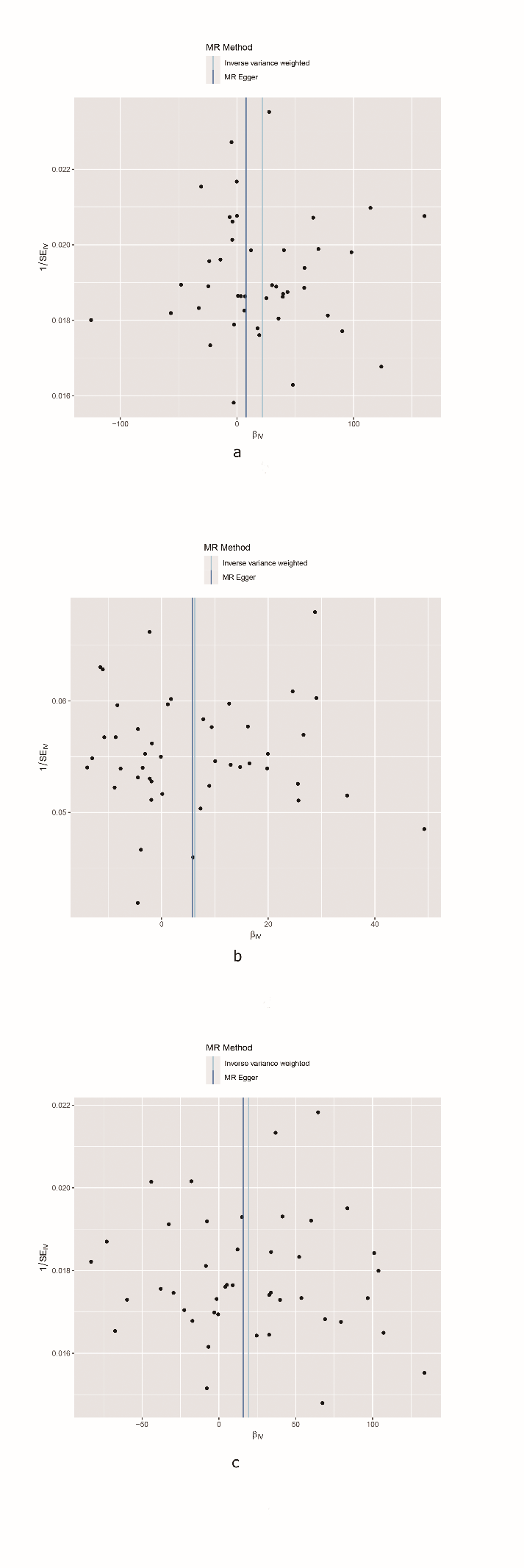


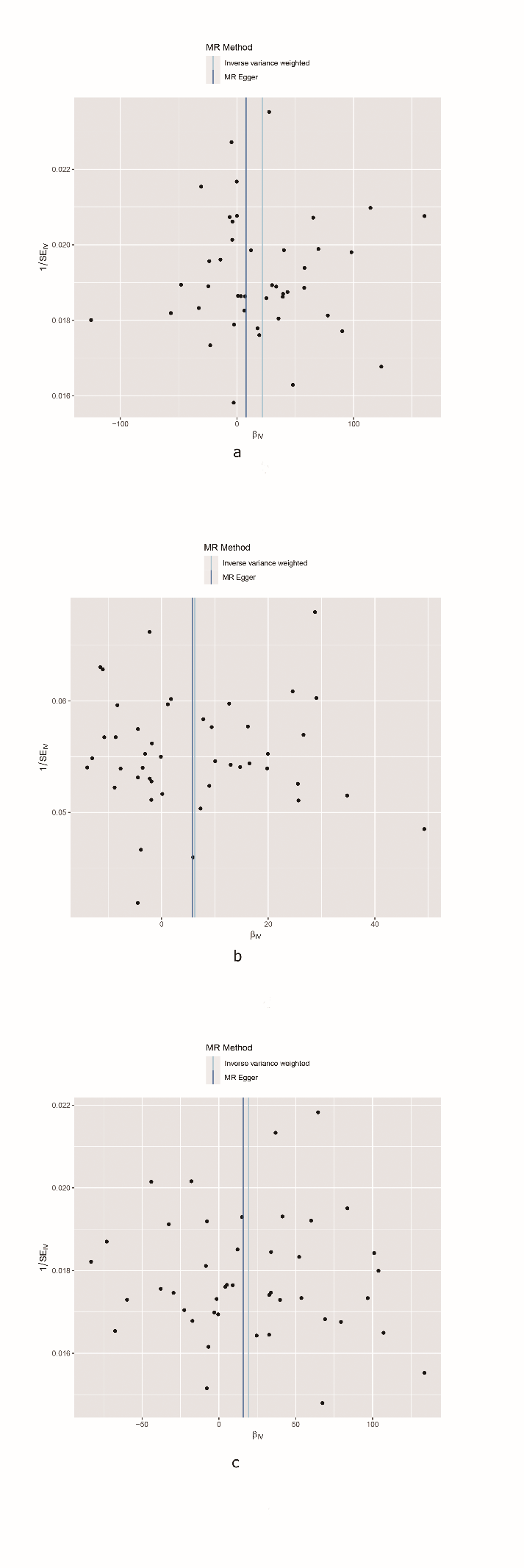

Supplement: Supplementary file 1 — Supplementary Material 1: Supplementary Figure 1. Local genetic covariance estimates of Heritability Estimation from Summary Statistics. Supplementary Figure 2. Forest plot of significant estimates identified with IVW. Supplementary Figure 3. Funnel plot from genetically predicted ASD on SA [file 12888_2024_5514_MOESM1_ESM.docx]
